# In Which Ways Does Community‐Based Dental Education Facilitate Development of Professional Identity in Undergraduate Curricula? A Scoping Review

**DOI:** 10.1111/eje.13084

**Published:** 2025-02-26

**Authors:** Sruthi Sunil, Juebei Chen, Kamran Ali, Trine Fink, Xiangyun Du

**Affiliations:** ^1^ College of Dental Medicine, QU Health Qatar University Doha Qatar; ^2^ Aalborg UNESCO PBL Centre, Department of Sustainability and Planning Aalborg University Aalborg Denmark; ^3^ Department of Health Science and Technology Aalborg University Aalborg Denmark

**Keywords:** community‐based dental education, outreach placement, professional identity, service learning, undergraduate dental students

## Abstract

**Background:**

Community‐Based Dental Education (CBDE) is becoming an integral part of undergraduate dental curricula globally. Literature has provided a wide range of benefits related to supporting the development of several professional skills such as communication and social interactions in community settings for dental students. While these skills are related to professional identity (PI) development, there has been little discussion linking CBDE with PI development in the current literature. To fill in this literature gap and explore pedagogical potentials to support PI for undergraduate healthcare curricula, this study provides a scoping review of the literature to analyse how published studies link CBDE to the development of PI.

**Methods:**

PRISMA extension for Scoping Reviews (PRISMA‐ScR) was used to conduct the review. The review protocol was registered with Open Science Framework to ensure transparency. Multiple electronic databases were searched, followed by screening of the articles based on the inclusion and exclusion criteria. Qualitative analysis of the included articles was conducted using NVIVO by two independent reviewers.

**Results:**

A total of 4483 articles were identified from databases. The full‐text screening was performed for 101 articles by two reviewers, out of which 35 articles were included in the analysis. The reviewed articles provide a diverse perspective of association and contribution to PI development through CBDE. Four dimensions were identified, including personal, clinical, interpersonal, and cultural. Most of the studies focussed on attaining clinical experience, while other studies explored confidence, teamwork skills, communication skills, and cultural awareness among undergraduate students in CBDE.

**Conclusion:**

The current study explores the value of CBDE to foster PI development in undergraduate education. Although the existing literature identifies some elements in CBDE that may contribute to PI development, the link between it and CBDE does not appear to be articulated clearly. The results of the review provide practical implications for the future practice of CBDE and its relevance to professional identity development.

## 1 Introduction

Community‐based dental education (CBDE) represents a structured learning approach wherein both community service and academic goals are pursued simultaneously. It fosters service learning, and active engagement within the community, and emphasises reflective practices [1]. Several studies have explored the impact of CBDE in undergraduate dental programmes and several benefits have been highlighted. The Commission on Dental Accreditation (CODA) states that ‘Dental education programs must make available opportunities and encourage students to engage in service‐learning experiences and/or community‐based learning experiences’ [2, 3]. CBDE has been shown to enhance students' skills in cultural competence, interprofessional collaboration, and practice management, as well as to benefit the school financially and address the oral health needs of diverse and underserved communities. Students who participate in CBDE provide a substantial amount of dental care for various patient groups, and they report feeling more comfortable in treating the under‐served populations [2]. Several factors have driven dental schools globally to implement various kinds of education models that include CBDE. CBDE involves both didactic teaching and practical learning in a community‐based dental setting [4]. In CBDE, students learn about the oral health issues of underserved and disadvantaged populations with poor access to dental care which allows the students to gain experience while benefitting the communities [5]. Dental students face several challenges while working in a community setting. Some factors may have a critical impact on students such as readiness to treat underserved populations, patient factors such as communicating with anxious patients, contextual factors like language barriers, and interpersonal factors like connecting with patients who may have limited trust in students' abilities [6].

Professional Identity (PI) is described as ‘the sense of being a professional’ [7]. PI in healthcare emerges when a member of a profession fosters the attitudes, beliefs, and standards that underpin the clinician's role leading to the development of an identity as a member within the profession along with a clear understanding of the responsibilities of being a health professional [7]. PI development in healthcare education usually begins with the start of the educational programme. It continues beyond graduation to adapt to the requirements of a rapidly evolving healthcare system. PI helps health professionals to define the scope and remit of their professional practice, identifying boundaries and in a clinical setting [8]. Literature in higher education (HE) emphasises the importance of the learning environment in fostering PI development. It is recommended to place PI as part of the core curriculum, to develop career aspiration and purposefulness among students. This includes connecting curriculum and work; implementing pedagogical strategies such as integrating professional practices into classrooms; active involvement and development of learner agency along with appropriate faculty support and mentorship; establishing a work‐like environment; and offering opportunities for professional socialisation [9].

CBDE has become an integral part of the student's learning experience and may contribute to PI development. PI development in dentistry can be linked to CBDE in several ways, such as opportunities for social interaction in the community setting and profession‐related skills (e.g. communication) [4]. Connecting the literature of PI and CBDE, a framework for conceptualising PI in relation to CBDE is proposed in this study, which views PI in three interrelated dimensions: (1) Personal values (self‐confidence, motivation, self‐image, career choice); (2) Clinical competencies (Hand skills, time management skills, clinical decision‐making skills); and (3) Interpersonal and socio‐cultural factors (team working skills, mentorship, institutional values and policies, site partnership and collaborations) [2, 10, 11].

First, regarding personal values, the literature highlights several elements in CBDE such as self‐image, confidence level, change in career choice support from mentors, teamwork skills, clinical competence, and development of hand skills which link with PI. CBDE not only serves to enhance student motivation to learn but also encourages them to take responsibility for managing their learning process. Embracing learners' subjective feelings about CBDE, behaviours, and interactions with the patients in a community‐based setting can impact positively on the PI of students.

Second, regarding clinical competencies, PI development within undergraduate healthcare curricula is a multifaceted process, influenced by the socio‐cultural environment along with academic and clinical exposure, expectations, and interaction with personal elements like values, beliefs, and responsibilities. It should also encompass active participation in the cultivation of professional skills through collaboration with the professional community and workplace [12].

Third, on the aspect of interpersonal and socio‐cultural factors, CBDE facilitates need assessment or situation analysis, solution finding, teamwork, and collaboration on project reports that demand both individual and group learner agency [13]. A study conducted by Du et al. (2022) assessed PI development among dental students in a unique pedagogical context, that is, problem‐based learning (PBL). The results emphasise the importance of PBL in developing PI while highlighting the challenges associated with a socio‐cultural framework along with pedagogical implications for future programme improvement [9]. In addition, students' PI development and CBDE experience are also impacted by their relations with mentors from their programme and the site partnership, as well as institutional policies [6]. Embracing and inculcating learners' subjective feelings about CBDE, behaviours, and interactions with the patients in a community‐based setting can impact positively PI development [12]. Although previous studies show that PI development among dental students may be influenced by social interactions in multiple learning settings including classroom, clinical, or community settings, the association between PI and CBDE has not been explored [9, 14].

With a growing interest in CBDE, a recent scoping review analysed evidence related to CBDE programmes in dentistry, exploring the aims of these programmes and determining how the learning outcomes of these programmes have evolved [1]. While the review provided multiple positive links between CBDE and student learning outcomes, such as assessing students' clinical treatment skills to ensure professional skills development, it did not explore the value of CBDE with PI development. Moreover, PI literature in dental education does not appear to explore how undergraduate dental students use their learning experiences to develop PI in a community settings. To address this gap in the literature and to explore pedagogical potentials for employing CBDE for PI development, this scoping review aims to provide an overview of how CBDE contributes to the development of PI in undergraduate dental curricula. The research question for this study was framed as follows: In which way does the current Community‐Based Dental Education (CBDE) support Professional Identity development among undergraduate dental students? The proposed framework serves as a foundation to conceptualise PI linking to a CBDE context and serves as a tool for data analysis.

## 2 Methods

### 2.1 Study Protocol and Registration

This scoping review adopted the updated methodological guidance proposed by the Joanna Briggs Institute (JBI) and reported by the Preferred Reporting Items for Systematic Reviews and Meta‐Analyses Extension for Scoping Reviews (PRISMA‐ScR) [15, 16]. The protocol was registered in the Open Science Framework [17].

### 2.2 Eligibility Criteria

The eligibility criteria were based on the PCC strategy with pre‐set inclusion and exclusion criteria as below:

- Population: Undergraduate dental students
- Concept: Professional Identity development
- Context: Community‐based dental education as part of the undergraduate dental curriculum

#### 2.2.1 Inclusion Criteria

The literature search focused on studies reporting on Community‐Based Education in a dental curriculum.

- Primary research studies on CBDE published in the last 31 years, that is, from January 1992 to December 2023
- Studies published in English

#### 2.2.2 Exclusion Criteria

- Opinion papers such as editorials, commentaries, reviews, or book chapters

### 2.3 Information Sources

A comprehensive search on electronic databases was conducted up to the last 31 years (January 1992– December 2023). Literature from relevant databases such as PubMed, Scopus, Embase, and ProQuest Central was included in the review.

### 2.4 Search Strategy

The search strategy comprised a combination of Medical Subject Headings (MeSH) and keywords for PubMed and index terms about the other databases, integrating Boolean operators to create meaningful search strings after consultation with an experienced librarian. The detailed search string is as summarised in Table 1.

**TABLE 1:** Keywords used to search electronic databases.

Concept 1	Concept 2
“Health education” [MeSH Terms] OR “Health Education, Dental”[Mesh] OR Dental school curriculum OR Dental program	Community‐based Dental Education OR “Public health dentistry”[MeSH Terms] OR CBDE OR Outreach placement OR Outreach program OR Service learning

### 2.5 Selection of Sources of Evidence

All the identified articles were imported to a reference management software (desktop version of EndNote version X9; Clarivate Analytics, London, UK). After the removal of duplicate articles, two reviewers screened the articles based on their titles and abstracts using Rayyan Systematic Review Screening Software [18]. The studies meeting the eligibility criteria and based on the themes of PI were selected for full‐text screening and the final selection. Any disagreements were resolved by consensus.

### 2.6 Data Charting and Coding Process

Data charting was done using Microsoft Excel and a codebook was created. Coding for the articles based on PI themes was performed using NVIVO Lumivero 14 software (Denver, Colorado, USA) [19]. The data items are summarised in Table 2.

**TABLE 2:** Themes for professional identity in community‐based dental education.

Author/Year of publication	Study methodology/no of participants	Personal	Clinical	Inter‐personal	Cultural	Study setting
		Confidence	Competency development	Teamwork	Cultural competency	
		Self‐image/Motivation	Clinical experience/Hand‐skills	Collaboration/site‐partnership	Cultural awareness	
		Career choice	Decision‐making skills	Communication with patients		
				Interaction with the supervisors (mentorship)		
1. Smith et al. (2009) [20]	Self‐reported survey Y 4 dental students at 2 intervals		Improvement in clinical skills (≥ 94%), Improvements in time management skills (95%)	Improvement in team‐working, patient management, and communication skills (≥ 94%)	Increased awareness of the primary care environment	Community Dental Service centres Sheffield UK
2. Abuzar et al. (2009) [21]	Quantitative—questionnaire survey for Y4 students	Practising in a rural area (≥ 75%)	Broad‐based clinical experience, boost in hand skills, and increased awareness of oral health issues in rural communities (≥ 30%)	Increased rapport with staff (~88.24%), mentorship from trained dentists, and collaboration with different government agencies	Increased understanding of indigenous issues in rural communities (~95%)	Rural Dental Rotations University of Melbourne, Australia
3. Bahammam et al. (2023) [22]	Quantitative –Pre‐test and post‐test questionnaire survey	Change in career choices after the rural postings (*p*‐value 0.004)	Significant improvement in intention to treat the low‐income patients (*p*‐value 0.04)	Improvement in connectedness with patients (*p*‐value 0.01)		External sites, Taibah University, Saudi Arabia
4. Altman et al. (2012) [23]	Quantitative‐ questionnaire survey for graduates of 2007 and 2010	Improved preparedness for a career in dentistry (≥ 95%)	Real‐life work experiences at multiple health Centres	Site partnership from different health centres		Service‐learning activities, University Arizona School of Dentistry, USA
5. Major et al. (2014) [24]	A quantitative study, of Year4 students who graduated in 2006 through 2011	Student's willingness to pursue specialty training in CBDE performed better (46%)	Better grades than the comprehensive course after CBDE rotations due to varied clinical exposure (≥ 50%)			CBDE rotation sites, University of Iowa, USA
6. Volvovsky et al. (2014) [14]	Self‐reported survey for dental students at the beginning and end of the term	Improved motivation to help underserved communities (< 0.001) Better connection between personal career interests and broader public goals		‐Increased interest in working with others to make a positive impact on the community, Opportunities to communicate with patients from diverse backgrounds	Greater recognition and respect for people from diverse backgrounds (< 0.001)	Community‐based activities, University of Michigan School of Dentistry, USA
7. Abuzar et al. (2016) [25]	Quantitative—questionnaire for final‐year students	Positive attitude to work in indigenous oral health settings		Effective assistance received by clinical supervisors and clinic staff	Increased understanding of the oral health needs of Indigenous (Aboriginal) issues	Clinical outplacements (outreach training, Melbourne Dental School, Australia)
8. Kuthy et al. (2010) [26]	Quantitative questionnaire survey, senior dental students 1992–2004	Willingness to treat low‐income population groups			Willing to treat groups that had low income and other ethnic groups (~60%)	University of Iowa extramural programme, USA
9. Mays et al. (2019) [27]	Self‐reported questionnaire survey, Y4 students (2016–2018)	Personal preference for future practice in rural settings (61.4%) (post‐rotation)				Clinical site for the University of Minnesota School of Dentistry USA
10. Goswami et al. (2017) [28]	Questionnaire survey 2010 to 2016, third to fifth‐year dental students	Student's confidence and (15%–30%)	Enhanced clinical experience and exposure to real‐life situations, a greater variety of patients, and time management skills	Team‐working skills with dental nurse and received faculty support to overcome the difficulties (15%)		Community Health Centre‐ Based Outreach Clinic, University Dental Clinic, Helsinki, Finland
11. Major et al. (2016) [29]	Self‐reported survey for Y4 students 2008 through 2014	Exhibited significant change in treating low‐income and specific populations after graduation (*p* < 0.05)	Improved recognition of responsibility to treat underserved populations (≥ 50%)	‐Clinical and faculty experiences positively influenced students' desire to treat underserved populations		University of Iowa College of Dentistry, community‐based clinics, USA
12. Mays et al. (2017) [30]	Self‐reported Y4 DDS students		Opportunities to provide preventive and operative treatments		Interactions with underrepresented minority communities and diverse patient groups.	Extramural clinics, University of Minnesota, USA
13. Bulgarelli et al. (2012) [31]	Qualitative interviews‐ Final year students	Increased self‐image and motivation to treat patients in different settings	Improved clinical decision‐making skills	Improved team‐working skills, Improved communication with patients	Improved respect for cultural differences	Outreach programme among a Brazilian indigenous community
14. Smith et al. (2006) [32]	Qualitative interviews	Career enhancing and a ‘real’ experience Enhanced motivation to consider outreach setting for their 1‐year vocational training	More clinical work is done in outreach settings than in hospital Experiential learning, patient management skills, and clinical decision‐making skills reinforced earlier theoretical learning	Sense of realism and being part of a dental team and appreciated the value of teamwork, Supervision, and mentorship provided, and a sense of freedom to practise in a controlled environment	A holistic approach to dentistry and the need to plan treatment compatible with patients' lifestyles and expectations	Outreach clinics across Northern England
15. Lynch et al. (2009) [33]	Qualitative study‐ Y4 dental students	Encouraged to think more for themselves and make decisions which increased self‐confidence. Feeling respected and improved self‐image	Increased clinical freedom and learning experience with a boost in confidence (≥ 80%)	Gaining the trust of supervisors Opportunities to interact with the dental team		Primary Care Unit, Cardiff University, UK
16. Smith et al. (2019) [2]	Quantitative questionnaire survey‐ US Dental schools	Developed professional responsibility (~70%)	Skills in Integrated practice management (~40%)		Develop cultural competency in diverse clinical settings (~93%), opportunities to interact with racial and ethnic minorities (~89.7%), and rural populations (~79.3%)	CBDE experiences, U.S. dental schools, USA
17. Eriksen et al. (2010) [34]	Qualitative study‐ Y4 autumn semester	Development of self‐confidence	Early insight into ‘real life’ experience/practice in different kinds of dental treatment procedures	Opportunities to learn teamwork and collaboration Interactions with supervisors		External University Clinics, University of Tromso, Norway
18. Leisnert et al. (2016) [35]	Self‐reported questionnaires in 2006 and 2010	Improved sense of responsibility and felt independent	Time management skills	Collaboration between the clinical mentor and the student was very high		Outreach dental education Malmo University, Sweden
19. Smith et al. (2022) [36]	Qualitative study‐ Final year between 2018 and 2019	Working in community‐based organisations (≥ 20%)	Opportunities to treat patients in a public health setting		Recognition of neglect of children, elderly, disabled, and culturally marginalised members of society	Clinical rotations in community‐based clinics, USA
20. Ahmad et al. (2020) [37]	Qualitative interviews, Y1 to Y5 dental students	Real‐world experience, self‐awareness, impact on personal outlook towards life and their perspective as a human being	Developed competencies in multiple aspects of patient care, communication with patients, organisational skills	Motivated to establish good dentist‐patient relationships	Socio‐culture diversity among visually impaired patients and	Dental students at a voluntary extramural educational programme, Kuala Lumpur, Malaysia.
21. Major et al. (2015) [24]	Qualitative questionnaire study	Prefer providing service for a high‐need population	Comfortable managing complex medical cases and developed clinical skills	Communicating with patients of different languages, working efficiently with the dental team CBCE faculty demonstrated how to perform specific procedures, more confidently	Providing dental care to ‘patients from underserved areas and multicultural backgrounds’	University of Iowa College of Dentistry, community‐based clinics, USA
22. Smith et al. (2019) [2]	Qualitative interviews‐ Fourth‐year students		Students were getting to do more procedures, different procedures than they might at school	Students get a different perspectives, tips, and strategies on how to do things from dentists in the real world	The benefit of partner sites' employing students upon graduation and indirectly as future advocates for community‐based dentistry Exposed to different cultures and they're trained in different ways of working with people who haven't been able to access care before	University of Illinois at Chicago, community‐based learning sites, USA
23. Mathieson et al. (2012) [38]	Qualitative data from 2009 and 2010 with focus group interviews for fourth‐year dental students	Development of self‐awareness and self‐confidence, interest in dentistry, or led to decisions regarding dentistry after graduating	Improvement in clinical decision‐making skills, knowledge about clinical environments, development as a professional, and experience of different clinical approaches. Real‐life experience in a controlled, school environment led to a clinical skill development	Communication with patients having phobia or anxiety and challenged to develop strategies for patient care Rotation sites focused on serving patients on publicly funded insurance plans	Improvement in cultural awareness especially while treating patients having language barriers	Community‐based clinic rotations, Arizona School of Dentistry & Oral Health, USA
24. Radford et al. (2016) [39]	Mixed methodology on dental students between 2014 and 16	Self‐confident and self‐awareness	Clinical decision‐making skills (80%)	Faculty and mentor support and advice to develop skills and good rapport with patients	Holistic approach treatment to socially disadvantaged	Outreach placement at the University of Portsmouth Dental Academy, UK
25. Joury et al. (2015) [40]	Mixed‐methodology, (short essay and a self‐completed questionnaire), 400 third‐year students	Personal growth compared to the traditional learning environment	Competency training developed independent learning compared to traditional dental settings (≥ 80%)	Communication skills with patients especially children with special needs		Outreach programmes, Damascus University, Syria
26. Hossain et al. (2022) [41]	Mixed methodology, final‐year undergraduate dental students	Self‐confidence as a dental practitioner (≥ 40%)	Improvement in clinical experience (~35%)			Urban‐based UQ and rural‐based LTU, Australia
27. Suresan et al. (2019) [11]	Experimental study design with postintervention questionnaire for 100 final‐year students	Appreciation of dental services, increase in self‐confidence. Identified personal strengths and weaknesses	Increase in hands‐on or experiential components (~52%)	Leadership, organisational, communication, and managerial skills by working together as a team. Partnership between institution and community	Development of cultural competencies by interacting (~39%) and communicating, public education to reduce oral health disparities	Rural outreach programmes, India
28. Decastro et al. (2003) [42]	Mixed methodology for senior dental students	Sense of responsibility, time management skills, and pursuit of community activities after graduation	Opportunity to gain more clinical training (*p* < 0.001) Gained experience working with fearful or anxious patients (~95%)	Work with dental auxiliaries (~93.5%)	Appreciation for cultural and social patterns affecting dental care (*p* < 0.05) and the diversity of the patient population	Community‐based clinic, New Jersey Dental School, USA
29. Mofidi et al. (2003) [43]	Mixed methodology‐ 160 senior students	Self‐awareness, caring for patients (≥ 50%) Self‐confidence (20%)	Enhanced awareness about the complexity of patients, ethical dilemmas, and complexity of dental care (≥ 30%)	Communication skills (~54%)	Awareness of macro‐level challenges (unemployment, lack of education, and racism)	Community‐based clinical rotation, University of North Carolina School of Dentistry USA
30. Rubin et al. (2008) [44]	Mixed methodology‐ dental students between 2003 and 07	Self‐confidence (> 0.5), Self‐motivated to learn languages to communicate	Helps prepare for the role of a dentist Motivated to treat patients with disabilities	Communication with patient or family members and the team members and collaboration with health care professionals	Influence of patient's cultural background/beliefs on dental treatment plan (> 0.5), Social responsibility regardless of the level of cultural competence exhibited upon entry into the programme	Community setting, Pittsburgh, USA
31. Holzman et al. (2008) [45]	Mixed methodology at 3 time points (freshman year—before, during, and after participation)	Developed personal efficacy (~17%)	Significant role in providing care for the needy (~20%)		Students' perceptions of their societal responsibility for the underserved remained stable	Outreach community setting, University of Southern California School of Dentistry, USA
32. Daher et al. (2011) [46]	Fourth‐year students‐ Mixed methodology	Motivated by the interest of the dentists and the team in having us there	Hands‐on skill development in paediatric dental treatment	The commitment of the outreach health team showed an association with the students' experience during all visits (*p* < 0.05)		Service‐learning programme, Dental School in Brazil
33. Sager et al. (2018) [47]	Mixed methodology‐ 108 senior dental students	Experience, positive influence towards public health dentistry, prefer to practise dentistry in a rural area	Professional responsibility is perceived as ethical and social responsibility		Professional responsibility to personally care for patients who are indigent in their community regardless of income status	Community‐based clinic rotations, University of Minnesota, USA
34. Rohra et al. (2012) [48]	Mixed methodology‐ 254 dentists participated in CBDE before graduating	The experience was a valuable part of education	Preferred treating patients in the outreach clinics than in the dental school clinics (~52%) Clinical skills and competency development (≥ 80%)	Faculty assistance in the community clinics was effective (≥ 70%)	Respect for people from diverse backgrounds other than my own (≥ 50%), treat more diverse patients	CBDE, University of Michigan, USA
35. Asgari et al. (2020) [49]	Mixed methodology, 120 senior (final‐ year)		Experience of oral health needs assessments and preventive oral health services (≥ 75%)	Inter‐sectoral interaction and communication		CBDE courses, Isfahan University of Medical Sciences, Iran

### 2.7 Data Items

The data items included the author's name, year of publication, title, study design, setting, and PI themes, and they were extracted by the first author.

### 2.8 Synthesis of Results

Another reviewer served as a second coder following a thematic approach combined with open coding and was given the codebook for extraction. This auditing process was done for validity. Inter‐rater reliability was also conducted on 10% of studies in this phase. IRR was 100%. The data were then extracted to a codebook independently by each coder using the same codebook. The independent coder was blinded to the data of the first author. Results were based on PI themes.

## 3 Results

### 3.1 Study Selection

**FIGURE 1:**
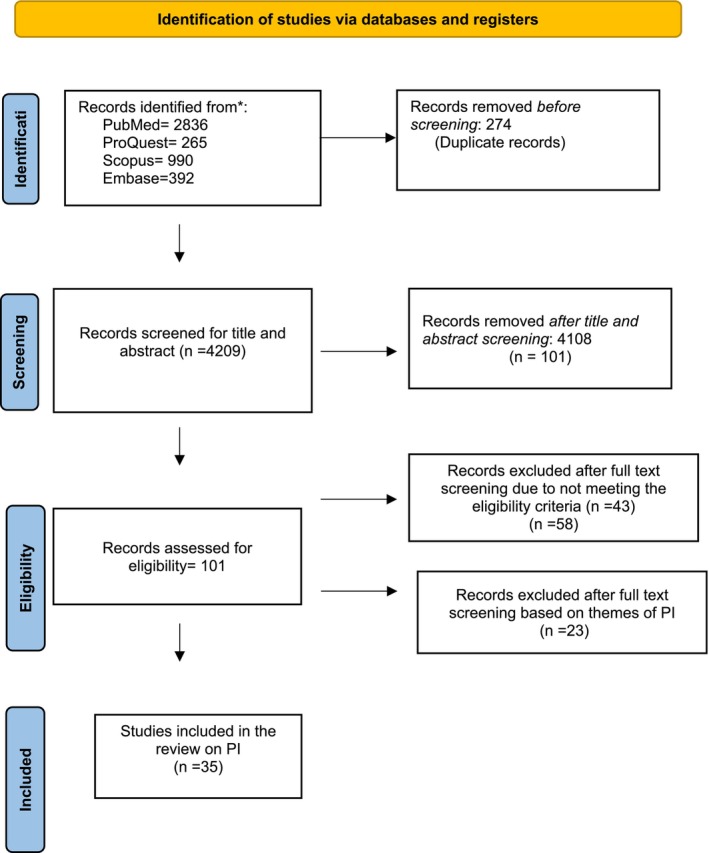
PRISMA flowchart of search results [15, 16].

The results of the literature search and study selection are shown in the PRISMA flowchart (Figure 1) as per PRISMA‐ScR guidelines [15, 16]. The total number of reports identified was 4483 articles from the selected databases. After the removal of 274 duplicate articles, 4209 articles were included for title and abstract screening. The full‐text screening was performed for 101 articles, out of which 66 were excluded for not meeting the eligibility criteria, that is, the components of PI were not identified. In all, 35 articles were included for qualitative analysis. Consensus was maintained throughout the selection of the articles and PI themes. A codebook was created based on PI themes, references, study setting, and the methodology used in the study, and participant information was extracted by the first author.

### 3.2 Characteristics of the Included Studies

An overview of the characteristics of the included studies in the context of PI development is summarised in Table 2. Of the 35 included studies, 12 used Qualitative methods, 11 Quantitative, 1 Experimental study, and 11 were Mixed‐methods studies. Considering the diversity of educational settings in different countries, it is important to consider contextual factors (study setting) that are important aspects while reporting the results [50]. All the studies mentioned the study settings, and the majority of the studies are conducted in the US (62%), UK (11%), and the remaining studies are from Australia, Sweden, Norway, Helsinki, India, and Iran.

### 3.3 In Which Ways Does CBDE Support PI Development?

This section reports the results of the integrated data analysis process. Following the proposed framework for conceptualising PI relating to CBDE as discussed in the introduction section, namely, three themes were explored in healthcare literature (personal, professional/clinical, and societal factors). Following this, an initial step of analysis identified characteristics of CBDE with the PI development according to these three dimensions. Subsequently, a bottom‐up thematic analysis identified an additional dimension, namely the cultural aspect, which has been recently well reported in CBDE literature and can be related to PI development. An overview of the four dimensions is presented in Table 2 and elaborated on in the following.

#### 3.3.1 Personal Dimension

The PI formation in the personal dimension is categorised by subthemes, namely motivation, confidence, self‐image, and career perception, as summarised in Table 2. The studies reported enhanced student motivation through their active engagement with the rural population [14, 51]. Positive perceptions towards choosing CBDE and willingness to work in a rural setting after their graduation following their outreach placements were also reported [25, 27, 47, 51]. Active engagement in CBDE activities was highlighted, and students were self‐motivated to treat underserved and minority populations by the end of their outreach placement programme [21, 35, 42].

#### 3.3.2 Clinical Dimension

Regarding the clinical dimension, the thematic analysis identified multiple benefits of CBDE. Firstly, studies across the board reported CBDE to be beneficial in developing clinical competency, clinical experience, and hand skills of students during their outreach placements, as seen in Table 2. Competency development, Increased awareness of the relative contributions made by the environment (physical, economic, and social), and patient management skills were also reported after rural placements. In addition, students also reported improved confidence in clinical decision‐making and time management skills [20, 21, 28, 35, 42]. Other profession‐related competencies were also addressed, such as dental services in community settings. This is an example of profession‐related competency [31]. Students also agreed that they were exposed to hands‐on or experiential components, and nearly half (48.53%) of the participants strongly agreed that outreach programmes should be implemented consistently in their curriculum, as they have better exposure to a wide clinical case mix [11, 24, 31, 32, 33].

#### 3.3.3 Inter‐Personal and Socio‐Cultural Dimension

Regarding inter‐personal aspects in CBDE, team working skills with the dental auxiliaries (~93.5%), as well as other members in the dental team, had considerably increased during their outreach placement [21, 42, 44]. A sense of realism was often associated with opportunities to communicate and build a rapport with patients with phobias, anxieties, child patients, and language barriers [32, 38, 40]. Students reported positive experiences about their interactions with mentors and faculty while treating the patients [33, 34, 35, 39]. There was collaboration/site partnership between institutions and different government agencies as well as within different communities [11, 21]. Students reported that participating in CBDE programmes made them more aware of their role as a dentist (35.3%) and enhanced their leadership skills (25.5%)in a post‐intervention questionnaire survey [11]. CBDE at Boston University Henry M. Goldman School of Dental Medicine, the senior students are expected to read the comprehensive extramural programme policies and procedures manual provided by the dental school before the students start their outreach placements, and the institutional policies are such that there is a formal agreement between the institution and the partner site, encompassing legal liabilities and no revenue‐sharing between the sites and the dental school [10, 52].

#### 3.3.4 Cultural Dimension

The cultural dimension is an additional finding from thematic analysis that is related to PI development. Studies addressed cultural awareness and cultural competency during engagements with rural and other indigenous populations as an important learning gain in CBDE, which adds to PI in a global context [2, 25, 30, 47]. Positive cultural experiences and competencies were seen among students undergoing CBDE programmes at various outreach centres. The Rural Dental Rotations (RDR) model equipped the students with a holistic approach when managing indigenous patients with underlying medical health issues. It has also provided opportunities to introduce students to the indigenous history and culture to which they would otherwise have little exposure [25]. The majority of the dental schools in the US recognise the importance of cultural and linguistic concepts that are included in the dental curricula [4, 52].

### 3.4 Synthesis of Results

The results indicate that the personal dimension, clinical, interpersonal, and cultural dimensions are an important aspect that supports PI development among undergraduate dental students in a professional setting. Most of the included studies are self‐reported by the undergraduate students (~70%). Out of which, some studies had empirical statements. The included studies offer diverse perspectives on viewing CBDE through the lens of PI development.

## 4 Discussion

The current literature in healthcare education underscores the need to focus on PI development to prepare future healthcare professionals for independent practice and services to the communities. To connect the concepts of PI and CBDE, A framework for conceptualising PI relating to CBDE is proposed in this study to understand PI development as a multifaceted, dynamic, interactive, and evolving process in CBDE. Results of the study suggest that these three dimensions are interactive and interrelated in CBDE literature. Personal factors such as self‐confidence, self‐image, and motivation are closely associated with clinical experience and communication skills [53]. Moreover, students' motivation and engagement can be influenced by relationships with mentors and collaborators in community settings [11, 41]. Additionally, CBDE can also facilitate cultural competence in students and can also contribute to PI development. Students must cultivate the ability to think and behave effectively within their professional roles in multiple cultural contexts. This entails understanding workplace dynamics in a multicultural community, meeting the expectations of patients, relatives, and colleagues, and collaborating efficiently with various stakeholders [53].

The current CBDE initiatives address PI development in positive ways, enabling students to gain real‐life experiences in a sociocultural context rather than solely focusing on clinical experience. However, PI development is a complex phenomenon, and the existing CBDE literature only addresses it partially. Social accountability is emerging as an important element of healthcare education and can be achieved through experiential learning, engagement with communities, and public and patient representatives to develop a collaborative approach to curriculum development [54, 55, 56].

Social accountability is now included explicitly in the recently published framework of behaviours and outcomes for dental professional education published by the General Dental Council (GDC), United Kingdom [57]. A clear and comprehensive understanding of social accountability is fundamental for a career in dentistry as clinical dental practice entails significant social interactions with the community [58]. Although social accountability did not emerge as a separate theme in the current review of CBDE, it relates closely to the interpersonal and socio‐cultural dimension of professional identity. CBDE offers numerous learning opportunities for students to develop social accountability, and this in turn can contribute to the PI of the future dental workforce. Like their peers in medical education, dental educators also need to consider educational innovation and research to develop and align assessment methods with teaching and learning related to social accountability [56].

Given the potential benefits of CBDE identified in this scoping review, it offers immense opportunities to facilitate PI development for undergraduate dental students. It is recommended that PI development be included as a learning outcome in undergraduate curricula and structured teaching on the importance of PI development may be provided to the students. These measures would enable educators and students to effectively utilise CBDE activities to enact PI from an early stage in the dental programme.

This study has a few limitations that need to be addressed. First, only key databases were included as part of the search strategy. This means some related articles may have been overlooked. Second, the included studies did not provide the demographics like age, gender, and socio‐economic status of the students that could have provided additional insights into the experiences of the students. We also found that none of the reviewed articles explicitly conceptualised professional identity but used the concepts as common terms. Future research should explore PI development in CBDE using mixed‐method research and multiple sources of data, not only quantitatively examining perceptions but also using qualitative methods to gain a deeper understanding of the value of CBDE in PI development among undergraduate dental students.

## 5 Conclusion

This paper may serve to sensitise the stakeholders about the linkages between CBDE and PI development. CBDE provides immense opportunities for dental students to interact with the community and can facilitate PI development. The findings of the current study suggest that while CBDE and PI development share some common goals, CBDE has not been utilised adequately to enact PI development. Appropriate measures are required to incorporate PI development in the learning outcomes of undergraduate dental curricula to facilitate the transition of the future dental workforce from the temporal confines of university settings into independent clinical practice.

## Author Contributions

X.D., K.A., and S.S. conceptualised the study. J.C. contributed as an independent coder. S.S., K.A., and X.D. conducted the data analyses; S.S., K.A., T.F., and X.D. contributed to drafting the manuscript; all authors reviewed and approved the manuscript.

## Ethics Statement

The authors have nothing to report.

## Conflicts of Interest

The authors declare no conflicts of interest.

## Data Availability

No new data were created during this study. Data sharing does not apply to this article.
